# A Rare Cause of Acute Pancreatitis in a Transgender Female

**DOI:** 10.1177/2324709620921333

**Published:** 2020-05-14

**Authors:** Lindsey C. Shipley, David T. Steele, Charles M. Wilcox, Chad M. Burski

**Affiliations:** 1 University of Alabama at Birmingham, AL, USA

**Keywords:** pancreatitis, estrogen, hypertriglyceridemia, transgender

## Abstract

Acute pancreatitis is defined as an acute inflammation of the pancreas and is most commonly caused by gallstones and alcohol followed by elevated triglycerides and medications. Estrogen as a cause of secondary hypertriglyceridemic pancreatitis is a rare but known phenomenon in females on hormonal therapy; however, it is not well described in the transgender female population. In this article, we present a case of a 31-year-old transgender female who developed acute, severe pancreatitis after a few months of using estrogen as transition therapy. To our knowledge, this is the third case report of a transgender female presenting with acute pancreatitis secondary to estrogen. Long-term supraphysiologic doses of sex hormones are required to maintain secondary sex characteristics placing this population at a higher risk of developing acute pancreatitis. Further research is needed to determine risk and screening methods to prevent this side effect.

## Introduction

Acute pancreatitis (AP) is defined as an acute inflammation of the pancreas with incidence varying between 4.8 and 24.2 cases per 100 000.^1^ Patients present with epigastric abdominal pain with radiation to the back, nausea, vomiting, and decreased appetite.^2^ Diagnosis is made by presenting with 2 of the 3 major criteria: characteristic epigastric pain, computed tomography (CT) evidence of AP, or elevated amylase or lipase 3 times the upper limit of normal.^3^ The most common causes of AP are gallstones and alcohol, followed by elevated triglycerides (TG) and medications. Hypertriglyceridemia (HTG) is defined by fasting serum triglyceride levels of >150 mg/dL. Hypertriglyceridemic pancreatitis is thought to be secondary to increased plasma viscosity from both an elevated HTG and chylomicrons leading to ischemia and resultant organ inflammation. Hypertriglyceridemic pancreatitis typically occurs in patients with a primary inherited dyslipidemia or secondary to uncontrolled diabetes, alcohol consumption, or medication use.^4^ Triglyceride-induced pancreatitis accounts for only 5% of cases but is responsible for half of all gestational pancreatitis cases.^4,5^ Estrogen-induced pancreatitis is well documented in females using hormone replacement therapy; however, to our knowledge, there are only 2 other reported cases of estrogen-induced pancreatitis in transgender females.^6,7^ In this article, we present a rare case of a transgender female who developed acute severe pancreatitis after a few months of using estrogen as sex change therapy.

## Case Report

A 31-year-old transgender female with no past medical history presented as a transfer after presenting to an outside hospital 2 weeks prior with AP. At the outside hospital, she had epigastric pain and laboratory workup significant for lipase >7500, TG >7000, and calcium of <4. She was initially managed conservatively but developed fevers with tachycardia prompting CT of the abdomen, which was significant for bibasilar pneumonitis with ascites and retroperitoneal fluid. A CT-guided drainage removed 650 cc of nonpurulent, old blood from 8 separate fluid collections. Fluid cultures were negative for infection. Despite negative culture data, including blood cultures and antibiotics (vancomycin, meropenem, and fluconazole), she continued to have high-grade fevers and tachycardia. She was ultimately transferred due to slight elevation in total bilirubin to 2.1 with cholelithiasis seen on CT scan for evaluation for magnetic resonance cholangiopancreatography versus endoscopic retrograde cholangiopancreatography.

**Figures 1 and 2.**
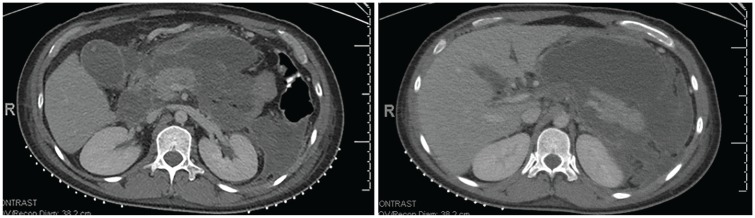
Computed tomography scan notable for a large peripancreatic fluid collection most consistent with walled-off necrosis.

On arrival, the patient reported fever, chills, muscle aches, severe generalized abdominal pain worse in epigastric region, and decreased appetite. She denied any past medical or surgical history. She was a former smoker and quit 1 year prior, would drink 5 to 6 alcoholic drinks per week with occasional “binge drinking,” and denied illicit drug use. She immigrated from Mexico several years prior and was transgender, identifying as a female. She denied family history of gastrointestinal disorders, HTG, or pancreatitis. Her only medication history was an occasional Advil or Tylenol as needed for headaches. Her vital signs were significant for a fever of 101.3°C, heart rate of 125 beats per minute, blood pressure of 117/64 mm Hg, oxygen saturation of 96% on room air, with a respiratory rate of 18 breaths per minute. She appeared uncomfortable and in moderate distress due to pain. She was tachycardic but with regular rhythm and without murmurs. Abdomen was soft, mildly distended with severe tenderness to the epigastrium without rebound. Bowel sounds were normal. There was no jaundice, rashes, or sclera icterus. Admission laboratory results were significant for a white blood cell count of 18 cells/L, hemoglobin/hematocrit of 8.7 g/dL, platelets of 793 000/µL, sodium of 132 mEq/L, albumin 2.8 g/dL, alkaline phosphatase 301 IU/L, total bilirubin 1.1 mg/dL, alanine transaminase 49 U/L, aspartate transaminase 55 U/L, and lactic acid 0.7 mmol/L. The serum concentration of TG had decreased to 345 mg/dL after 2 weeks of cessation of estrogen therapy and supportive care at the outside hospital. Right upper quadrant ultrasound showed no evidence for biliary obstruction, dilation, or cholelithiasis. In-house interpretation of the outside hospital CT scan was notable for a large peripancreatic fluid collection most consistent with walled-off necrosis (Figures 1 and 2). At this point, the most likely diagnosis was AP due to very severe HTG. However, it was unclear why this patient would have such severe levels of TG. She did not have any known family members with HTG to suggest a genetic cause and did not have a history to suggest recent alcohol use, diabetes, hypothyroidism or medication-induced. On further questioning, the patient reported that she had purchased and started taking oral estrogen therapy 2 months prior from an online vendor to transition from male to female, the likely cause of her very severe HTG. She was unaware of the formulation or dose of the estrogen.

Initial management included aggressive intravenous fluid resuscitation at 250 cc/h and antibiotics, vancomycin, meropenem, and fluconazole. Infectious workup included negative blood cultures, urine cultures, lower extremity ultrasound with Dopplers, chest X-ray, human immunodeficiency virus, gonorrhea, and chlamydia. Interventional radiology was consulted who placed 3 percutaneous retroperitoneal drains in the fluid collections. As the fluid collections decreased in size, the patient began to symptomatically improve. The patient was continued on vancomycin and meropenem to complete a 2-week course. Two weeks after discharge, the patient had IR drain exchange, and repeat CT scan showed almost complete resolution of the fluid collections and her drains were removed. The patient did not seek further treatment for transition therapy due to this acute illness.

## Discussion

Estrogen is a rare cause of drug-induced pancreatitis with ~40 cases reported worldwide and only 2 published case reports in transgender females^6-8^ (Table 1). With a growing number of transgender patients presenting within the health care system (9.2 per 100 000) and transgender females requiring supraphysiologic doses of estrogen therapy to maintain secondary sexual characteristics, it is important to understand their risk for and ways to prevent AP.^9^

**Table 1.** Case Reports of Transgender Females With Estrogen-Induced Pancreatitis.

Study	Age (Years)	Peak Triglyceride Level	Length of Therapy	Complications
Perego et al^8^	37	5174 mg/dL	3 months	Severe necrotizing pancreatitis, retrocavity fluid collections, and bilateral pleural effusion
Goodwin et al^7^	51	2073 mg/dL	10 years	Acute interstitial pancreatitis
Our patient	31	>7000 mg/dL	2 months	Severe necrotizing pancreatitis, bilateral pneumonitis, and retroperitoneal fluid collections

Estrogen levels during pregnancy decrease the activity of lipoprotein lipase and increase the levels of very low-density lipoprotein leading to decreased catabolism of serum chylomicron and triglyceride concentrations. It is proposed that exogenous estrogen cause HTG by a similar mechanism; however, the level of evidence is lacking as it has only been reported in case reports. The excess free fatty acids and elevated chylomicrons are suspected to increase plasma viscosity and thus lead to ischemia and organ inflammation of the pancreas.^4^ The most common offenders are oral contraception, hormone replacement therapy for menopausal symptoms, and in vitro fertilization-embryo transfer.^10^ Consequently, hormone supplementation with estrogen or tamoxifen is not recommended when serum TG levels are >500 mg/dL.^11^ The initial presentation of HTG-AP is similar to that of AP of other causes; however, a detailed history should be obtained in those presenting with HTG-AP and include secondary causes such as obesity, alcohol abuse, uncontrolled diabetes, hypothyroidism, chronic renal failure, and medications such as corticosteroids, retinoids, and estrogen.^12^ Physical examination findings that can help narrow the diagnosis are eruptive xanthomas on extensor surfaces, lipemia retinalis, signs of metabolic syndrome, and hepatosplenomegaly from fatty infiltration of the liver.^13^ Patients with HTG pancreatitis may have a worse outcome than patients with other types of pancreatitis^13^ and HTG levels >500 mg/dL may cause falsely normal amylase levels.^14^ It is unclear whether severity of pancreatitis is directly associated with degree of TG elevation. In a study looking at patients with familial pancreatitis, there was a correlation with increasing TG; however, other smaller case studies did not correlate.^12,15^ Furthermore, it is not known whether there is an increased risk with a specific route, dose, formulation, or duration of therapy. In the previous 2 case reports, one patient was taking 2 mg of estradiol twice a day for 10 years, while the second patient was taking a combination of estrogenic and antiandrogenic compounds (conjugated estrogens 0.625 mg, cyproterone 2 mg, and ethinyl estradiol 0.035 mg) for 3 months.^7,8^ Prevention requires a goal TG level of <200 mg/dL with the use of a fibrate, niacin, or n-3 fatty acids either alone or in combination. For patients with hyperchylomicronemia, a diet that is restricted from fat and simple sugars is recommended.^11^ Initial management includes aggressive intravenous fluid resuscitation, initial bowel rest, and pain control, as well as discontinuing estrogen therapy and lowering TG levels. Lowering TG to <500 mg/dL has been shown to expedite clinical improvement. No other definitive guidelines exist for the management of severe HTG, but multiple treatment modalities have been described using apheresis, insulin, and heparin.^16^ If a patient has concomitant hyperglycemia, intravenous insulin can be considered.^16^ If not, then apheresis can be considered if well-tolerated.^17^ The primary aim is to prevent pancreatitis from becoming necrotizing and prevent organ failure. For long-term therapy, an antihyperlipidemic agent is recommended, such as gemfibrozil 600 twice per day along with dietary fat restriction (<15% daily caloric intake from fat).^18^

## Conclusion

In transgender patients considering or currently using estrogen therapy, consider obtaining a baseline lipid panel pretherapy and yearly lipid panels after initiation of therapy to identify those at high risk for dangerous side effects, such as AP. If baseline TG level is >500 mg/dL, consider not initiating estrogen therapy or start a triglyceride lowering medication and closely monitor.
